# The Vividness of Happiness in Dynamic Facial Displays of Emotion

**DOI:** 10.1371/journal.pone.0026551

**Published:** 2012-01-11

**Authors:** D. Vaughn Becker, Rebecca Neel, Narayanan Srinivasan, Samantha Neufeld, Devpriya Kumar, Shannon Fouse

**Affiliations:** 1 Cognitive Science and Engineering, Arizona State University, Mesa, Arizona, United States of America; 2 Department of Psychology, Arizona State University, Tempe, Arizona, United States of America; 3 Centre of Behavioural and Cognitive Sciences, University of Allahabad, Allahabad, India; University of Leuven, Belgium

## Abstract

Rapid identification of facial expressions can profoundly affect social interactions, yet most research to date has focused on static rather than dynamic expressions. In four experiments, we show that when a non-expressive face *becomes* expressive, happiness is detected more rapidly anger. When the change occurs peripheral to the focus of attention, however, dynamic anger is better detected when it appears in the left visual field (LVF), whereas dynamic happiness is better detected in the right visual field (RVF), consistent with hemispheric differences in the processing of approach- and avoidance-relevant stimuli. The central advantage for happiness is nevertheless the more robust effect, persisting even when information of either high or low spatial frequency is eliminated. Indeed, a survey of past research on the visual search for emotional expressions finds better support for a happiness detection advantage, and the explanation may lie in the coevolution of the signal and the receiver.

## Introduction

One of the most cited ideas in the emotion perception literature is that angry faces “pop out” of crowds—that they can be detected equally rapidly regardless of the number of other faces in the crowd [1], [2]. From one perspective, this effect makes adaptive sense, because rapidly detecting facial cues of impending interpersonal violence would facilitate the avoidance of said violence. On the other hand, however, the most successful ancestral attackers were likely those that concealed their threatening intentions, which likely would have selected *against* a vivid display of anger [3]. Furthermore, a growing chorus of voices in cognitive science question whether previous demonstrations of an anger superiority effect (ASE) might not reflect idiosyncrasies of the stimuli used in particular experiments [4]–[6]. Critically, the stimuli that show the ASE are often static schematic images only slightly more complicated than the iconic smiley face, and are thus susceptible to the criticism that equally simple low-level perceptual features drive the detection asymmetries [7]. For example, if a schematic angry face has more angular features, and if feature detectors of the visual cortex detect angularity faster than curviness, such stimuli would give rise to an apparent anger advantage even in subjects who were not attending to the emotion of the display. Indeed, one stimulus set (used in [2]) has been used to show anger detection advantages in subjects with autism [8] as well as in elderly subjects [9], despite the fact that these populations generally have more difficultly processing emotional expressions. This raises the possibility that these results may simply show that basic feature detectors—which are more plausibly still intact in these participants—are readily activated by the features of the schematic stimulus, and *not* that expressions of anger have been preferentially detected.

Clearly, support for the claim that angry faces are more efficiently detected requires stimuli that are more ecologically valid. Unfortunately, more realistic faces often give rise to a happiness detection advantage relative to both angry [4], [10] and sad [11] faces. In fact, even schematic happy faces are better identified in a flanker task relative to schematic angry/threatening faces [12]. In short, the expression detection literature is not only inconsistent in its conclusions, but also rife with criticisms that particular effects arise only from idiosyncrasies of unrealistic and ecologically-invalid visual displays.

It is surprising, then, that most of this work uses *static* facial displays of emotion—the more realistic experience of seeing a face become angry or happy has received almost no attention. Given that effective social communication often depends on the need to quickly detect the dynamic aspects of expressive change, it is important that researchers begin to fill in this gap in the literature. Horstmann & Ansorge [13] made a laudable effort in this regard (and failed to find an ASE that was not confounded with other display attributes), but they did not use real faces. Therefore, in this paper, we conduct four experiments using dynamic changes in expression from neutral to happy or angry. We investigated both identification performance with singleton faces as well as a single changing expression in the context of multiple faces (as in visual search tasks). In addition, we also investigated the role of spatial frequency information in the identification of dynamic changes in expression, which has recently been shown to enhance the detectibility of both positive and negative images.

## Methods

### General Method and Materials

**Figure 1:**
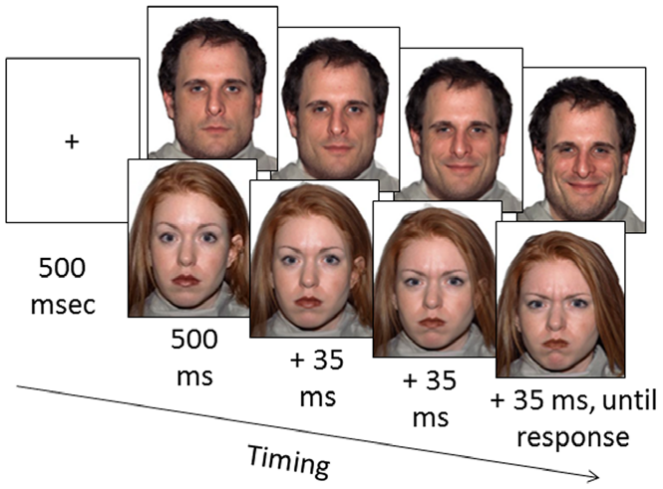
Two possible morph sequences, timed to give the impression of a video clip in real time (endpoint images adapted from Tottenham, et al., 2009; the people displayed provided consent for publication of the photos in publications and on the web).

To explore the detection of dynamic expressions of anger and happiness, we first selected photographs of actors portraying closed-mouth expressions of anger, neutrality and happiness from the MacBrain stimulus set [14]. Using closed-mouth expressions had the advantage of eliminating certain high-contrast features in the lower part of the face, particularly the exposed teeth of a full smile, a feature that has confounded many previous demonstrations of happiness detection advantages (e.g., [10]). For each actor, we then created a morph-continuum running from the neutral exemplar to each expressive extreme. Presenting static images from this continuum in a rapid sequence (see Figure 1) generated the appearance of a video clip of a person becoming angry or happy. One advantage of this approach was that the timing of the dynamic change was precisely controlled and was made up of components that changed in a linear fashion, maximizing the realism of the expressive dynamics without sacrificing the ability to equate the onset and offset of the timing.

The faces selected included both African and Caucasian male and female faces. Using the Nimstim naming conventions, we selected individuals 1, 5, 7, 9, 11, 12, 13, 14, 20, 21, 22, 23, 39, 40, 41, and 43. Pilot work confirmed that subjects perceived these to be realistic video recordings—none suspected that we were showing them a succession of morphs.

Across experiments we used the software package DirectRT to display the experimental stimuli and collect reaction times. In all experiments, participants provide informed consent and then sat approximately 60 cm away from a flat screen monitor in a cubicle, and decisions were recorded by pressing keys on the computer keyboard.

#### Ethics Statement

This research was approved by the Arizona State University Institutional Review Board, and all participants read and signed statements of informed consent.

### Experiment 1

We first investigated emotion identification of singleton faces to see whether happy or angry emotional changes are detected better.

#### Participants

Seventy-eight subjects participated, but ten exited the program before all of the data were collected. Only the 68 participants with complete data were retained for the analysis.

#### Procedure

In this first Experiment, participants were presented with fairly large (6×9 cm) displays of these dynamic expressions, presented one at a time at the center of a computer screen. There were 96 trials, with faces presented in a randomized order. Participants had to rapidly indicate that the face was angry or happy as soon as the expressive change was apparent to them.

#### Experiment 1 Results

Participants were 37 ms faster identifying the change to a happy expression (M = 453 ms; SD = 86 ms) than the change to anger (M = 490 ms; SD = 122 ms), *t*(67) = 3.30, *p* = .0016. Thus, in spite of the fact that more muscles are recruited by anger than by happiness, happiness appears to involve the more vivid expressive change.

Because each face appeared bearing both dynamic expressions over the course of the trials, we also conducted a dependent samples t-test with faces as the unit of analysis, and confirmed that the happiness advantage was significant, *t*(15) = 4.91, *p*<.0001 (in the experiments that follow, such item analyses are not reported but were routinely consistent with subject analyses). There were no differences for accuracy. Follow-up analyses did not reveal any other factors or interactions that compromised the conclusion that happy expressions were better identified than angry expressions. The happy advantage is consistent with previous research using static images of anger and happiness [4], as well as findings using happy and sad faces [15]. The results of Experiment 1 raise questions about the studies that claim superiority of detection of negative emotional expressions especially given the more ecological and dynamic nature of the present stimuli.

### Experiment 2

Experiment 1 presented a promising finding, but the majority of demonstrations that facial displays of anger are more detectible come from searches for expressions in *crowds* of faces [1], [2]. This “visual search” paradigm affords stronger inferences about whether stimuli more quickly draw attention to their location, and so may be better suited to reveal an adaptive advantage for angry faces.

#### Participants

Although 45 subjects participated, 5 were removed for abnormally low accuracy (less than 2.5 SD below the grand mean) and one participant was eliminated for exceptionally long reaction times (more than 3 SD above the mean).

#### Stimuli and Procedure

Because we wanted to show each stimulus in each location more than once without vastly increasing the number of trials, for this experiment we used only the four white male stimuli (20, 21, 22, 23) from the first study, which resulted in a total of 192 trials (admittedly, this is a small number of stimuli, but we wanted to ensure that no location by identity interaction could compromise the results; Furthermore, we should note that almost every past demonstration of the ASE used a single angry—and generally schematic—stimulus, so our four exemplars afford more generalizability than most past studies—see [4] for a more rigorous survey of the previous studies and their shortcomings). Participants were told that they would see either a single face at the central fixation point or four faces (of different identities), one in each quadrant of the screen (each at an equal distance—approximately 5 cm—from the central fixation point). The task was to attend to and identify the expression as rapidly as possible with a key press. We assessed detection efficiency as a function of whether peripheral faces showed up on the left or the right; past work suggests that this may moderate the detection of positive and negative signals [11], [16].

**Figure 2:**
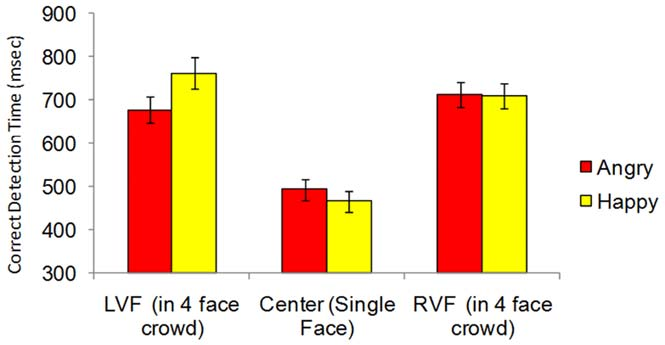
Reaction times to correctly identify the stimulus as a function of the type of dynamic expression (becoming angry vs. becoming happy) and its location. Standard error bars are included to provide a sense of variability across subjects, but do not correspond to the within-subjects hypothesis tests reported in the text. *Experiment 2 Results.*

Figure 2 shows the result of Experiment 2. When faces were presented on their own in the center of the display, we found a 28 ms advantage (SD = 81 ms) for happy faces, *t*(38) = 2.13, p = .04, replicating experiment 1. When the faces were presented in the periphery, however, there was no expression advantage in the right visual field (*t*<1), while in the left visual field, there was an 85 ms (SD = 112 ms) *advantage for angry faces*, *t*(38) = 4.74, p<.0001. It bears emphasizing that these were exactly the same faces, appearing on the left, right and center. Contrasting the detection of the same expression across right and left locations showed that angry faces presented on the left were detected 36 ms (SD = 100) faster than on the right, *t*(38) = 2.24, p = .031. Moreover, happy faces presented on the left were detected 53 ms more slowly (SD = 137 ms) than on the right, *t*(38) = −2.40, *p* = .021. In other words, presentation in the LVF improved the detectibility of angry faces while it hurt the detectibility of happy faces.

This lateralization effect is consistent with previous research showing that the right hemisphere of the brain—which receives visual input from the LVF—shows a specialization for processing information that we want to avoid [17], while the left hemisphere—receiving input from the RVF—is specialized for approach-related emotions and stimuli (note here that while the experience of anger may be an approach–related emotion [18], the angry *face* is a stimulus that we likely want to avoid).

### Experiment 3

While these peripheral results are intriguing, location is confounded with perceptual load, because the peripheral faces only appear within 4-face crowds, while the central faces appear alone. To verify that these lateralization effects generalized to single presentations, Experiment 3 was conducted.

#### Participants

Although 59 subjects participated, 2 were removed for abnormally low accuracy (less than 2.5 SD below the grand mean) and one participant was eliminated for exceptionally long reaction times (more than 3 SD above the mean).

#### Stimuli and Procedure

This study replicated the design of Experiment 2, but also included trials in which a single neutral face appeared in one of the peripheral locations, which increased the number of trials to 240. Upon appearing, the peripheral expressions immediately began to transform to anger or happiness (i.e. 35 ms after the neutral face onset, it was replaced by a slightly expressive image). This was necessary because any single brief onset stimulus automatically grabs attention [19], and we wanted to ensure that the expressive dynamics were underway before the person made a saccade to the stimulus location. We also reincorporated the White female stimuli used in study 1 (images 1, 5, 7, 9), to broaden the selection of items and ensure that expression lateralization effect was not contingent on masculine gender. Note however, that every face and expression combination appeared in every location for every subject.

#### Experiment 3 Results

Experiment 3 again revealed a 36 ms (SD = 70 ms) advantage for detection of happy faces vs. angry faces when targets were presented at the center of the display, *t*(55) = 3.82, p = .0003. The lateralization benefit was again seen for angry targets, which showed a significant left-side advantage in both crowds (M = 27 ms, SD = 106 ms), *t*(55) = 1.88, p_one-tailed_ = .032, and when appearing on their own (M = 40 ms, SD = 113 ms) *t*(55) = 2.66, p_one-tailed_ = .005. Peripheral happy targets showed evidence of a nonsignificant trend for a right-side advantage when embedded in crowds, *t*<1, but did show a significant advantage when presented peripherally on their own (M = 30 ms, SD = 129 ms) *t*(55) = 1.72, p_one-tailed_ = .046.

These results suggest that perceptual load cannot account for the lateralization effects. We should be wary of adaptive explanations for this result, however, because any plausible adaptation for detecting peripheral anger (or happiness) should have produced equivalently fast detections regardless of location. This result instead provides new evidence for a hemispheric asymmetry in approach vs. avoidance processing. In contrast, the happiness detection advantage at the central (foveated) location may suggest a legitimate adaption at the level of the signal design: Happy faces have a form that is more detectible. Because this form emerged via natural selection, it suggests that the facial display of happiness is a social signal that wants to be seen, and seen rapidly, and accordingly, it has appropriated signaling qualities that make use of basic feature detectors in order to maximize the likelihood that its prosocial message gets through. It follows then, that the happiness advantage should be robust to stimulus degradation.

### Experiments 4a & 4b

**Figure 3:**
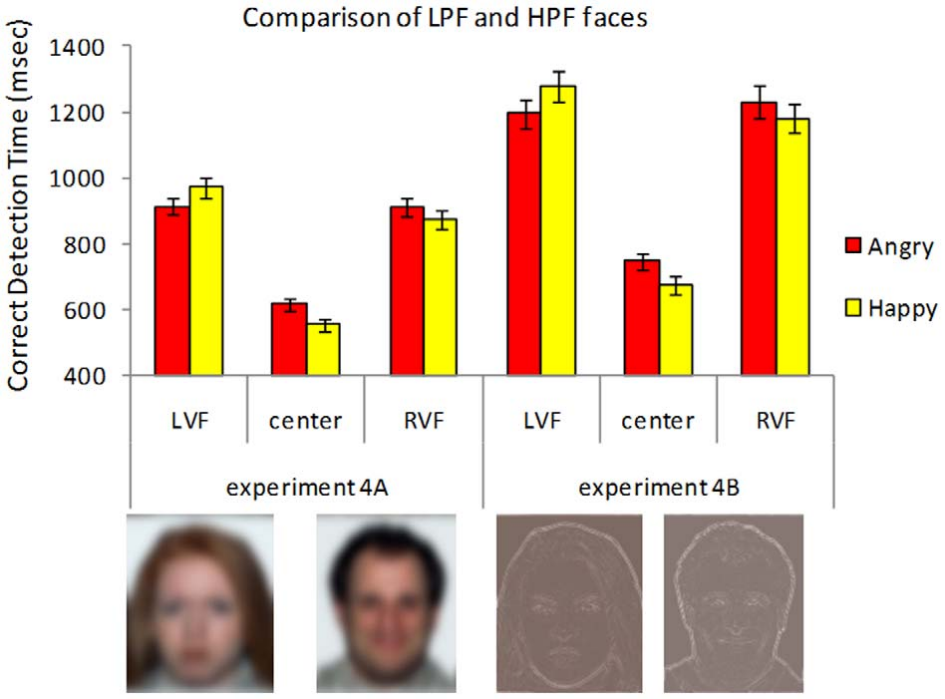
Reaction times to correctly identify the stimulus as a function of the type of dynamic expression (becoming angry vs. becoming happy), its location, and whether it was high or low pass filtered. Standard error bars are included to provide a sense of variability across subjects, but do not correspond to the within-subjects hypothesis tests reported in the text.

One factor that has been shown to differentially influence the detection of positive and negative stimuli is the spatial frequency of the information presented [20]. Fourier analysis can be used to decompose visual images into their component spatial frequencies, and consequently filter out all of the high frequency information—the sharp lines and contours that convey much of the detail of images—leaving a low pass filtered image that is a very blurry replica of the original (see Figure 3). Researchers have found that threatening images show a detection advantage that persists after Low Pass Filtering (LPF) [20], and have claimed that a fast subcortical route to the emotional centers of the brain processes this coarse threat-relevant information, an adaptation that enables us to rapidly respond to threat.

Recently we (DK & NS) have shown that the removal of low spatial frequency information significantly decreased the speed at which static happy expressions were identified [15]. In contrast, filtering out low frequency information with a High Pass Filter (HPF) benefits the detectibility of negative (sad) expressions relative to happy expressions. HPF may therefore wipe out the happy face advantage at central locations. If, however, the happy expression evolved to vividly and unambiguously signal positive affordances, we might expect to see the advantage for dynamic happiness persisting across both low and high pass filtering.

#### Participants

Experiment 4A included 84 subjects, but 8 were removed for low accuracy (less than 2.5 SD below the grand mean). Experiment 4B included 86 participants, but 4 were removed for error rates<−2.5 SD below the grand mean and 1 was removed for mean current reaction times >3 SD above the mean.

#### Stimuli and Procedure

For the image filtering in this pair of experiments, we used a Gaussian filter. The low pass filter cutoff was 3.33 cycles per degree, which amounted to 8 cycles per face; the high pass filter cutoff was 13.13 cycles per degree or 32 cycles per face.

The design consisted of two replications of Experiment 3, substituting dynamic LPF faces in Experiment 4a and dynamic HPF faces in Experiment 4b—in all other respects the designs were exactly the same.

#### Experiment 4a & 4b Results

The results in this pair of experiments provided consistent support for the happy face advantage, and showed a robust lateralization effect for happy faces as well (we collapse over single vs. multiple peripheral conditions, for ease of interpretation). In Experiment 4a, centrally presented LPF dynamic expressions of happiness were detected 60 ms faster (SD = 85) than their similarly filtered angry counterparts, *t*(75) = 6.18, p<.0001. There was no difference in the speed with which angry faces were detected when they appeared on the left vs. the right, *t*<1. There was, however, a big reaction time difference for happy faces, with those on the right detected 101 ms faster (SD = 136 ms) than those on the left, *t*(75) = 6.53, p<.0001. This happy face asymmetry consists of both a left-side cost—relative to LVF anger , a 69 ms slow-down (SD = 185 ms) in response times, *t*(75) = 3.28, p<.001—and a right side benefit—relative to RVF anger, a 35 ms facilitation (SD = 106 ms) in response times, *t*(75) = 2.90, p = .002.

In Experiment 4b, centrally presented HPF dynamic expressions of happiness were detected 68 ms faster (SD = 176 ms) than their similarly filtered angry counterparts, *t*(80) = 3.49, p<.001. There was no difference in the speed with which angry faces were detected when they appeared on the left vs. the right, *t*<1. There was again a big reaction time difference for happy faces, with those on the right detected 120 ms faster (SD = 251 ms) than those on the left, *t*(80) = 3.7, p<.001. Again, this happy face asymmetry is a consequence of both a left side cost—relative to LVF anger, a 99 ms slow-down (SD = 316 ms) in response times, *t*(80) = 2.83, p = .006—as well as the suggestion of a right side benefit—relative to RVF anger, a 39 ms facilitation (SD = 106 ms) in response times, *t*(75) = 1.54, p = .125. The lateralization advantage for angry expression compared to the happy expression in the left visual field is consistent with other findings that show advantage for negative emotions in the LVF/right hemisphere [16]. The laterality effect is much more pronounced in the right hemisphere compared to the left hemisphere.

## Results and Discussion

The present research produced two principal effects. First and foremost, the consistent advantage for detecting happiness at the focus of attention does appear to speak to the adaptive properties of this signal. Indeed, the fact that these advantages persist even for LPF and HPF images suggests that the dynamics of the happy expression have evolved to better appropriate the processing efficiencies of the human visual system at a number of different levels. For example, becoming happy involves an expansion of the face while becoming angry involves a contraction, and psychophysical work has determined that expansion is more efficiently detected than contraction [21] (perhaps because expansion is something that approaching, *looming* objects do, see [22]). To be clear, we are not postulating that the human perceptual apparatus evolved to be on the look-out for happiness (although there may be a perceptual readiness for the receiver to detect it as well), but rather that the form of the expression evolved to appropriate pre-existing efficiencies in the visual system. Although facial displays of emotion are ancient signals, they are not eternal—human facial expressions have evolved as signals in a coevolutionary “arms race” with perceptual receivers, and here both the signaler and the receiver benefit from the rapid detection of prosocial (or submissive) intentions. Indeed, even in chimpanzees, bared-teeth displays are now thought to communicate benign intent and function to reduce uncertainty rapidly in both aggressive and affiliative interactions [23].

We should also note that the changes in expression in our dynamic images occur fairly rapidly and it is plausible that the pathways that are sensitive to high temporal frequencies or changes would respond to this change in expression. In terms of visual pathways, the magnocellular pathway is more sensitive to high temporal frequencies and also low spatial frequencies. The consistent advantage for dynamic happy faces indicates that they might be subserved by the magnocellular pathway. This is also consistent with experimental results using static faces which indicate the importance of low spatial frequencies in detecting happy expression [15]. While this is hypothetical, it provides a plausible neural substrate for a happy face advantage that may have evolved for better social communication.

The second principal finding is that whether these expressions appear on the left or the right has a significant impact on their detectibility. This is consistent with properties of hemispheric specialization that have already been suggested in the literature [17], but ultimately does not reveal much about the adaptive design of expression perception. However, the results are fairly consistent with other findings using static emotional faces indicating a preference for negative expressions by the left hemisphere. We also find a bias for happy expression in the left hemisphere. If there is evolutionary advantage for detecting happy expressions (in social communication), then perhaps that might have become linked to the language specialization in the left hemisphere. It is also possible that approach emotions are linked with speech acts and hence might underlie a left hemispheric bias.

### The vividness of happy facial expressions in the broader literature

Our results may come as a surprise to many, for as we noted at the outset, the belief that angry faces are efficiently—and even preattentively—detected is widespread. Indeed, two of the more prominent studies [1] & [2] have each been cited over 400 times. A careful examination of the literature supporting the ASE, however, shows a problematic tendency to rely on simple schematic line drawings of anger and/or single target faces used repeatedly over hundreds of trials; both of these design features (or rather flaws) make it likely that participants learn to use idiosyncratic stimulus elements to perform the detection task without emotion perception coming into play at all. Horstmann and colleagues (e.g., [13]) have done an admirable job of experimentally demonstrating the shortcomings of various schematic stimuli. In contrast, when a variety of more realistic and ecologically valid photographic images are used and participants actually have to attend holistically to the emotional expressions to perform the task, happiness is more rapidly and accurately detected (see [4], for both a review of the literature and experimental evidence for this contention). We therefore feel confident that when the empirical findings are weighted by the ecological validity of the experimental designs, there is overwhelming evidence that happy faces are detected more efficiently than angry faces. We call this a *vividness* effect because we believe that the signal has evolved a detectable form in the same way that, for example, the black and yellow stripes of a hornet evolved to make use the perceptual mechanisms of potential predators and other threats. But we should be careful to note that these vividness effects occur early in perception (and possibly without the conscious application of attention), and that at later stages of information processing we may well see advantages for angry faces. For example, once seen and attended, angry faces may hold on to that attention and resist attentional disengagement (e.g. [24]). Thus, while happy faces are vividly detected, angry faces may be quite vivid once attended and in memory.

### Conclusion

These results should compel cognitive scientists to begin thinking about what facial expressions evolved to signal, and the costs and benefits of the signals' detectibility. Expressions of happiness convey approachability, friendship, possibilities for affiliation, trade and coalition building, and the sight of a happy face can de-escalate tension, all of which has caused the facial display of happiness to converge on salient and detectible forms (indeed, Hagar and Ekman [25] made a similar case about happiness over 30 years ago). Expressions of anger, on the other hand, have less reason to be salient at the earliest stages of visual perception. Sometimes anger communicates frustration and strong disapproval with the aim of holding attention. But if the expression accompanies a desire to attack another, a visually salient facial display of rage is only adaptive if its perception causes the target to back down; if there is no opportunity to preempt physical conflict, if one must aggress against another who is relatively equal in power/ability, then concealing one's intention—masking one's anger—is more beneficial [3]. It is therefore difficult to make the case that angry facial expressions would have evolved a form that could draw attention to their location, because the advantage to the perceiver is balanced by the cost to the displayer, and so the selective pressure would not promote visual salience. Communicating happiness, on the other hand, benefits the perceiver and the displayer, and would be expected to converge on forms and dynamics that are clearly and rapidly detected.

The present results thus exemplify a more principled approach to emotional signal detection that takes into account the ecologically valid form of the signal as well as the design of the receiver. It also represents one of the first explorations of the detectibility of dynamic facial expressions of emotion. We hope these results spur similar advances in theorizing and research, because until cognitive science wrestles with the coevolved nature of social signals and their perceivers, it provides an incomplete picture of why the mind works the way that it does.

## References

[1] Hansen CH, Hansen RD (1988). Finding the face in the crowd: an anger superiority effect.. Journal of Personality and Social Psychology.

[2] Öhman A, Lundqvist D, Esteves F (2001). The face in the crowd revisited: a threat advantage with schematic stimuli.. Journal of Personality and Social Psychology.

[3] Fridlund AJ (1994). Human facial expression: An evolutionary view.

[4] Becker DV, Anderson US, Mortensen CR, Neufeld S, Neel R (2011). The face in the crowd effect unconfounded: Happy faces, not angry faces, are more efficiently detected in the visual search task.. Journal of Experimental Psychology: General.

[5] Horstmann G (2009). Visual search for schematic affective faces: Stability and variability of search slopes with different instances.. Cognition and Emotion.

[6] Hunt AR, Cooper RM, Hungr C, Kingstone A (2007). The effect of emotional faces on eye movements and attention.. Visual Cognition.

[7] Horstmann G (2007). Preattentive face processing: What do visual search experiments with schematic faces tell us?. Visual Cognition.

[8] Ashwin C, Wheelwright SJ, Baron-Cohen S (2006). Finding a face in the crowd: Testing the anger superiority effect in autism.. Brain and Cognition.

[9] Mather M, Knight M (2006). Angry faces get noticed quickly: Threat detection is not impaired among older adults.. Journal of Gerontology: Psychological Sciences.

[10] Juth P, Lundqvist D, Karlsson A, Öhman A (2005). Looking for foes and friends: Perceptual and emotional factors when finding a face in the crowd.. Emotion.

[11] Srivastava P, Srinivasan N (2010). Time course of visual attention with emotional faces.. Attention, Perception, & Psychophysics.

[12] Srinivasan N, Baijal S, Khetrapal N, Srinivasan N, Kar B, Pandey J (2010). Effect of emotions on selective attention and control.. Advances in Cognitive Science: Volume 2.

[13] Horstmann G, Ansorge U (2009). Visual search for facial expressions of emotions: A comparison of dynamic and static faces.. Emotion.

[14] Tottenham N, Tanaka J, Leon AC, McCarry T, Nurse M (2009). The NimStim set of facial expressions: judgments from untrained research participants.. Psychiatry Research.

[15] Kumar D, Srinivasan N (2011). Emotion perception is mediated by spatial frequency content.. Emotion.

[16] Baijal S, Srinivasan N (2011). Emotional and hemispheric asymmetries in shifts of attention: An ERP study.. Cognition & Emotion.

[17] Davidson RJ (1988). EEG measures of cerebral asymmetry: Conceptual and methodological issues.. International Journal of Neuroscience.

[18] Coan JA, Allen JJB (2003). Frontal EEG asymmetry and the behavioral activation and inhibition systems.. Psychophysiology.

[19] Muller HJ, Rabbit PMA (1989). Reflexive and voluntary orienting of visual attention: Time course of activation and resistance to interruption.. Journal of Experimental Psychology: Human Perception and Performance.

[20] Mermillod M, Droit-Volet S, Devaux D, Schaefer A, Vermeulen N (2010). Are Coarse Scales Sufficient for Fast Detection of Visual Threat?. Psychological Science.

[21] Takeuchi T (1997). Visual search of expansion and contraction.. Vision Research.

[22] Schiff W, Caviness JA, Gibson JJ (1962). Persistent fear responses in rhesus monkeys to the optical stimulus of ‘looming’.. Science.

[23] Waller BM, Dunbar RIM (2005). Differential behavioural effects of silent bared teeth display and relaxed open mouth display in chimpanzees (Pan troglodytes).. Ethology.

[24] Fox E, Russo R, Dutton K (2002). Evidence for delayed disengagement from emotional faces.. Cognition and emotion.

[25] Hager JC, Ekman P (1979). Long-distance transmission of facial affect signals.. Ethology and Sociobiology.

