# Correction: The Vividness of Happiness in Dynamic Facial Displays of Emotion

**DOI:** 10.1371/annotation/f0519e8c-f347-4950-b7e8-3e9cbc3ec2a9

**Published:** 2012-05-23

**Authors:** D. Vaughn Becker, Rebecca Neel, Narayanan Srinivasan, Samantha Neufeld, Devpriya Kumar, Shannon Fouse

There were errors in Affiliations of the third author, both in the PDF and the online version. The correct Affiliations for Narayanan Srinivasan is 3. Centre of Behavioural and Cognitive Sciences, University of Allahabad, Allahabad, India.

